# Culturally competent patient–provider communication in the management of cancer: An integrative literature review

**DOI:** 10.3402/gha.v9.33208

**Published:** 2016-11-30

**Authors:** Ottilia Brown, Wilma ten Ham-Baloyi, Dalena RM van Rooyen, Colleen Aldous, Leonard Charles Marais

**Affiliations:** 1 School of Clinical Medicine, University of KwaZulu-Natal, Durban, South Africa; 2 Department of Nursing Science, Nelson Mandela Metropolitan University, Port Elizabeth, South Africa; 3 Faculty of Health Sciences, Nelson Mandela Metropolitan University, Port Elizabeth, South Africa

**Keywords:** cultural competence, patient–provider communication, cancer, oncology, culture

## Abstract

**Background:**

Managing cancer in a multicultural environment poses several challenges, which include the communication between the patient and the healthcare provider. Culture is an important consideration in clinical care as it contributes to shaping patients’ health-related values, beliefs, and behaviours. This integrative literature review gathered evidence on how culturally competent patient–provider communication should be delivered to patients diagnosed with cancer.

**Design:**

Whittemore and Knafl's approach to conducting an integrative literature review was used. A number of databases were systematically searched and a manual search was also conducted. Specific inclusion and exclusion criteria were set and documents were critically appraised independently by two reviewers. Thirty-five documents were included following these processes. Data extraction and synthesis followed and were also independently verified.

**Results:**

Various strategies and personal characteristics and attitudes for culturally competent communication were identified. The importance of culturally competent healthcare systems and models for culturally competent communication were also emphasised. The findings related to all themes should be treated with caution as the results are based mostly on low-level evidence (Level VII).

**Conclusions:**

More rigorous research yielding higher levels of evidence is needed in the field of culturally competent patient–provider communication in the management of cancer. Most of the available literature was classified as non-research evidence. The themes that emerged do, however, provide some insight into how culturally competent patient–provider communication may be delivered in order to improve treatment outcomes in patients diagnosed with cancer.

## Introduction

Communicating with cancer patients can be challenging for healthcare providers. The life-threatening nature of the illness, the physical and psychological suffering of cancer patients (1), and the responsibility of conveying complex health information to the patient while also managing their emotions (2) are just some of the challenges impacting patient–provider communication in the cancer setting. Managing cancer in a multicultural context further complicates patient–provider communication (3). Culture can be defined as ‘a system of beliefs, values, rules and customs that is shared by a group and is used to interpret experiences and direct patterns of behavior’ (4). Culture plays a significant role in how patients’ health-related values, beliefs, and behaviours are shaped (4) and affects how patients and communities approach the diagnosis and treatment of cancer as well as their trust towards healthcare providers and institutions (5). Similarly, culture has been shown to affect professionals’ and institutions’ approach to minority patients (5) and contributes substantially to the existing disparities in access to healthcare for minority and underprivileged patients (1). An example is South Africa as this country presents with disparities in health and wealth that are amongst the highest in the world (6). The majority of the South African population is classified as African (80.5%) (7) and consists of a number of ethnic groups each with their own African language. South Africa has 11 official languages comprising various African languages, English, and Afrikaans. African patients understand health and illness within a framework of indigenous beliefs which takes the biological, social, emotional and spiritual aspects into account and where cancer may be conceptualised as resulting from witchcraft or conflicts in relationships. Consultation with traditional healers is thus often preferred to Western medicine (8). Late presentation of cancer patients due to a preference for traditional approaches to healing has been reported in local and international studies (8–10). In addition, consultation with family members and the elders is a common practice before any major life decisions (8), including treatment decisions like surgery, are made. South African patients presenting for treatment in the public health sector tend to be confronted with cultural and language discordant medical encounters as healthcare providers are often not of the same cultural background as the patient; may have more urban, Western perspectives of health and illness; and are trained in English or Afrikaans. Similar reflections regarding culturally discordant medical encounters are noted in international literature where countries such as the United States and the United Kingdom serve populations from diverse cultural backgrounds (11–13).

Cultural competence has been proposed as a strategy to improve access to healthcare and the quality of healthcare, and to reduce and/or eliminate health disparities (14–20). Cultural competence has varied definitions (1, 17, 21–27) but seems to require the acquisition, integration, and application of awareness, knowledge, skills, and attitudes regarding cultural differences in order to effectively deliver expert care that meets the unique cultural needs of patients; to manage and reduce cross-cultural misunderstanding in discordant medical encounters; and to successfully negotiate mutual treatment goals with patients and families from different cultural backgrounds. Surbone (1) suggested that culturally competent cancer care can improve treatment outcomes and viewed cultural competence as a requirement for healthcare professionals working in the cancer setting.

Reviewing the literature revealed that there were no systematic or integrative reviews available on culturally competent patient–provider communication with cancer patients. This integrative literature review is part of a broader study for developing an evidence-based practice guideline for culturally competent patient–provider communication with patients diagnosed with osteosarcoma in a specific South African context. Healthcare providers in cross-cultural clinical settings have to be able to communicate an understanding of patients’ cultural beliefs while at the same time communicating the urgency of intervening and the effect on survival if patients choose to delay intervention while engaging in cultural practices. This integrative literature review aims to provide some insight into how to deliver culturally competent patient–provider communication to adult patients diagnosed with cancer.

## Methods

An integrative literature review was performed in accordance with the guidelines provided by Whittemore and Knafl (28). These authors propose the following key stages: problem identification, literature search, data evaluation, data analysis, and data presentation. This literature review methodology was selected as it allows for the inclusion of studies with diverse methodologies, and for the combination of data from theoretical and empirical literature, to facilitate a more comprehensive understanding of a particular issue or healthcare problem (28). The review question was formulated using the PICO guide (29). The aim of the integrative review was to determine how culturally competent patient–provider communication is best delivered to adult patients diagnosed with cancer.

### Literature search

**Fig. 1:**
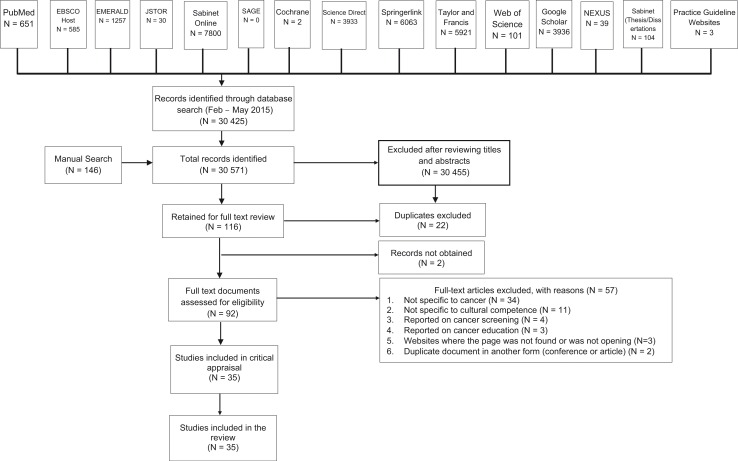
PRISMA flowchart detailing flow of studies through the review.

An experienced librarian assisted the primary author with selecting the keywords and databases, and with conducting the search. In the period February to May 2015, various electronic databases as is depicted in Fig. 1, were searched. Evidence-based practice guideline websites were also searched, including the National Guideline Clearinghouse, National Health and Medical Research Council (NHMRC), New Zealand Guidelines Group (NZGG), Registered Nurses’ Association of Ontario (RNAO), The National Institute for Health and Care Excellence (NICE), Scottish Intercollegiate Guidelines Network (SIGN), eGuidelines, Guidelines International Network, Turning Research into Practice (Trip) Database, Canadian Medical Association (CMA) Infobase, and Joanna Briggs Institute (JBI) Evidence-based Practice Database. A manual search was conducted using Google Scholar and the following websites: (www.culturediversity.org/cultcomp.htm, www.diversityrx.org/HTML/DIVRX.htm, www.omhrc.gov/templates/, www.sis.nlm.nih.gov/outreach/multicultural.html, www.npin.cdc.gov/pages/cultural-competence, www.adventisthealthcare.com/health/equity-and-wellness/, www.hrsa.gov/culturalcompetence/qualityhealthservices, www.cancer.gov/aboutnci/organization/crchd,iccnetwork.org/pocketguide/, www.cdc.gov/cancer/healthdisparities/statistics/ethnic.htm).

The following keywords were used in various combinations to conduct the literature searches: patient–provider communication; doctor–patient communication; physician–patient communication; cancer; oncology; cultural competence; culturally competent communication; cross-cultural communication; multicultural communication; and transcultural communication. Various sets of keywords were used that were deemed suitable for the databases, to ensure that no relevant literature was missed.

### Inclusion and exclusion of records

The following inclusion criteria were used: relevant literature from 1982 was included, as the term ‘cultural competence’ first appeared in the literature in 1982 (30). The literature on cultural competence had to pertain specifically to cancer or to cultural aspects of communication in the context of cancer care, and had to be available in English. Owing to the paucity of research documents available on the topic, non-research documents were also included when these were appraised as relevant to the review question (31).

Regarding exclusion of records, literature that pertained to cultural competence in disciplines other than the context of cancer care was excluded from the review. Literature pertaining to paediatric oncology, cancer patient education not related to the interaction between patients and healthcare providers, and cancer screening were also excluded. Inclusion and exclusion of records was independently verified by the second author using the inclusion and exclusion criteria. Figure 1 represents the search process for this integrative literature review.

### Data evaluation

A comprehensive and frequently used hierarchy system (Table 1) was chosen to rate the evidence (32).

**Table 1:** Rating system for the hierarchy of evidence for intervention/treatment questions (32)

Level I (strongest evidence)	Evidence from a systematic review or meta-analysis of all relevant randomized controlled trials (RCTs)
	Evidence from evidence-based clinical practice guidelines based on systematic reviews of RCTs
Level II	Evidence obtained from at least one well-designed RCT
Level III	Evidence obtained from well-designed controlled trials without randomization (quasi-experimental study)
Level IV	Evidence from well-designed non-experimental studies (case–control, correlational, cohort studies)
Level V	Evidence from systematic reviews of descriptive or qualitative studies
Level VI	Evidence from a single descriptive or qualitative study
Level VII	Evidence from the opinion of authorities and/or reports of expert committees

Critical appraisal tools were used to carefully and systematically examine the records in order to judge its trustworthiness, and its value and relevance in a particular context (33). The primary author and other authors independently appraised the documents.

Two quantitative studies were appraised using the Health Care Practice Research and Development Unit (HCPRDU) Evaluation Tool for Quantitative Studies (34). Four qualitative studies were appraised using the Critical Appraisal Skills Programme (CASP) tool for assessing qualitative research (35). Non-research records (*N*=29) were appraised using the Johns Hopkins Nursing Evidence-based Practice tool for Non-Research Evidence Appraisal (31). After critical appraisal was done, all 35 records were included for data extraction and synthesis.

### Data analysis

Data relevant to the review question were extracted from the included records. The primary author conducted the data extraction and content analysed the extracted data. The second author independently verified both processes in order to improve the rigour of the data analysis. Data display matrices were developed to facilitate data comparison and synthesis. The researchers employed an iterative process by repeating the data extraction and synthesis numerous times, in order to ensure the verification of the results.

## Results

The 35 records that met the inclusion criteria are presented in the Supplementary file. Two of the records could be classified as level IV evidence, 8 as level VI evidence, and 25 as level VII evidence. Six themes emerged from the data extraction and synthesis. Skills that healthcare providers require for culturally competent communication was the most prominent and most densely represented theme in the literature (*N*=32), followed by healthcare provider awareness (*N*=24), healthcare provider knowledge (*N*=22), culturally competent healthcare systems (*N*=22), personal characteristics and attitudes (*N*=13), and models for cross-cultural communication (*N*=3). Themes are discussed and summarised in Table 2 in the order of frequency with which they appeared in the literature. The literature referred to a range of healthcare professionals, including oncologists, surgeons, and nurses, but most of the sources did not specify the type of healthcare professional involved; hence, the term healthcare provider is used generically.

**Table 2:** Themes and subthemes

Themes	Subthemes	Delineation of the subthemes
Healthcare provider skills (*N*=32)	Communication skills (11, 18, 36) (*N*=18)	Engage in: • culturally sensitive communication (1, 5, 43, 49) • culturally congruent communication (5, 11) • clear (11, 44), accurate (11, 44), open (39, 42, 51, 53), flexible (42), and transparent (51) communication • congruent verbal and non-verbal communication (5, 11) Deliver care that patients understand (43, 57) Deliver culturally and linguistically sensitive services (55) Provide information in the patient's language (44) Learn the language (44) Develop a vocabulary of terms familiar to the patient (44) Provide clear information (48) Use simple language (38–42) Encourage the patient to ask questions (48) Repeat explanations (41) Check patients’ understanding of information (36, 39–41, 43, 44) Observe culturally appropriate non-verbal communication etiquette (5)
	Managing difference in the patient–provider encounter (*N*=13)	Avoid stereotyping and generalisations (5, 11, 37, 18, 36, 44, 45) Treat patients equally (38, 42) Avoid making assumptions about patient race, nationality, and language (1) Encourage patients to raise concerns about discrimination (39) Create a culturally safe and caring environment (41, 53) Individualise patient care (45, 53)
	Skills required for building the patient–provider relationship (*N*=12)	Invest time in the beginning (36, 46, 47) Engage the patient (11) Build rapport (41, 48) Gain patient trust (11, 17, 42, 48, 49) Actively engage patient in decision-making (36, 43, 48) Encourage and empower patients to raise trust issues (39) Address patients according to cultural preference (11) Recognise inherent power differentials (11) Be open about own cultural frame of reference (59) Acknowledge own cultural background to patients (1) Respond skilfully to cultural discordance (11)
	Ability to conduct a patient assessment (18, 26) beyond the biomedical (*N*=11)	Assess patients’ specific communication needs (46) Conduct a cultural assessment (40, 46, 51) • Active exploration of cultural issues (54) • Invite patients to describe their cultural backgrounds (57) • Explore views on family and community in the healthcare context (57) • Explore cultural (11) and health beliefs (54) • Explore family expectations, feelings, and concerns (51) • Explore level of family involvement required (54) • Determine who the main decision-makers are (patient or family?) (45, 47) • Explore preferences for truth disclosure (1, 36, 54) Conduct a spiritual assessment (51) Explore religious beliefs (1)
	Accommodating the patient's family (*N*=5)	Invest in and gain family trust (11, 38) Communicate with extended family as per patient's directive (11) Balance autonomy and dependency when meeting patient and family needs (52) Afford the family maximum control possible if this is a patient need (51)
	Accommodating religion and spirituality (*N*=4)	Recognise patients’ spiritual needs (53) Acknowledge the role of religion in the patient's belief system (11, 42) Demonstrate respect for religious beliefs (38)
Healthcare provider awareness (*N*=24)	Contextual awareness (*N*=11)	Awareness of: • country's socio-political history (41) • socio-cultural factors that affect the patient–provider relationship (45) • patients’ different phases of acculturation to the dominant culture (47, 56) • patient demographics in the service area (54) • the role of gender and religion in culture (48, 52) • patients’ level of education (38, 42) • patients’ experiences of discrimination in clinical settings (39) • dominant cultural narratives regarding health and illness (59) • culturally constructed myths about cancer (49) • patients possible combining allopathic and traditional medicine (59)
	Self-awareness (1, 18, 26) (*N*=9)	Awareness of own: • culture (55) • cultural beliefs (19) • belief systems (54) • spirituality (51) • own cultural assumptions, biases, and stereotypes (5, 18, 45, 54, 55)
	Interpersonal awareness (*N*=5)	Interpersonal awareness of: • inherent power differentials between patient and provider (41) • interaction between patient and provider's culture (40, 55) • communication differences between cultures (36, 48)
	Awareness of cultural expectations in the healthcare setting (*N*=5)	Awareness of: • the level of family involvement required (44, 54) • the role of family in cross-cultural clinical settings (1, 44, 52, 56) • patients’ possibly expecting a directive approach from providers (54)
Healthcare provider knowledge (*N*=22)	Context specific knowledge (*N*=9)	Knowledge of: • cultural groups attending services in the provider's clinical setting (11, 18, 36, 47, 52, 57) • serviced population's disease profiles, health disparities, and treatment outcomes (36, 37) • cultural health-related needs and health-seeking behaviours (18) • cultural approaches to illness and treatment (45) • cultural meanings of cancer (5) • patients’ perception of their illness (36) • influence of culture on how patient interacts with healthcare system (54)
	Self-knowledge (*N*=6)	Knowledge of own: • culture (11, 18, 36, 59) • belief system (18) • own biases and stereotypes (5, 11, 18, 54)
	Knowledge of patient's culture (*N*=5)	Knowledge of patients’: • health belief systems (11, 39, 44) • traditional health system (44) Knowledge of: • the role of gender and family in the decision-making (44, 47) • preferences regarding language used to discuss cancer (1) • non-verbal communication standards (1)
	Knowledge of broader contextual factors (*N*=5)	Knowledge of: • racism, sexism, and ageism (19, 62) • socio-political barriers to accessing healthcare (5, 11, 18) • the impact of past and present racism (18) • the role of gender, age and role expectations in the communication process (5) • socio-historical cultural context (5) • socio-cultural differences between self and patient (18)
Culturally competent healthcare systems (*N*=22)	Characteristics of culturally competent healthcare systems (*N*=6)	• are responsive to individual needs and to how cultures are perceived (18, 49) • promote and facilitate effective patient-centred communication (18) • respect cultural differences, and support effective care for diverse populations (18) • provide ethnic-specific services (5) • convert an awareness of disease prevalence into practices and policies (37) • develop and implement policies to support effective cross-cultural communication (18, 53) • link with culturally competent agencies and community organisations that provide bilingual and bicultural navigation, promotions, and community health outreach services (5) • have adequate support services (53)
	Strategies employed by culturally competent healthcare systems (*N*=17)	• use patient navigators (11, 24, 47, 48, 60, 61) • use professional translators (1, 5, 17, 39, 44, 45, 48, 54, 57, 59) • use of culturally sensitive print, visual, and audio-visual media and electronic communication (39, 43, 48) • use images to assist providers when discussing cancer with patients (41) • monitor patient characteristics (39) • translate written communications (45) • provide language-concordant encounters (39) • provide patient-centred care (60) • consult communities on cultural needs (41) • integrate community resources into cancer care (5) • display images of people from cultural groups attending the service (41) • have ethnically similar staff visible (41)
Healthcare providers’ personal characteristics and attitudes (*N*=13)	Healthcare providers’ personal characteristics (*N*=11)	• individual sensitivity (1, 18, 46, 61) • humility (1, 62) • empathy (18, 41, 57, 62) • curiosity (62) • compassion (18) • sincerity (19, 60) • tolerance (19) • acceptance (53) • authentic and respectful at all times (11, 19, 53, 57, 60) • value others (53)
	Healthcare providers’ attitudes (*N*=13)	• take responsibility for cultural aspects of health and illness (45) • take responsibility for combating discrimination in healthcare settings (45) • willingness to learn from patients (11) • openness to change and growth (53) • cultural sensitivity (1, 45, 53) • willingness to listen (53) • respect for cultural diversity, for the patient's culture and their cultural values (5, 11, 39, 42, 45, 52, 54, 62) • appreciation of different health belief systems (62) • willingness to explore culture with individual patients (36) • validate different cultures (57) • continual self-examination and self-reflection to examine one's own values and assumptions (18, 19, 53) • willingness to adjust behaviour and attitudes (36) • reflection on own interaction with cultural groups in the clinical setting (36)
Models of effective cross-cultural communication (*N*=3)	Kleinman's questions (17, 57) (*N*=2)	What do you think has caused your problem? Why do you think it started when it did? What do you think your sickness does to you? How severe is your sickness? Will it have a short or long course? What kind of treatment do you think you should receive? What are the most important results you hope to receive from this treatment? What are the chief problems your sickness has caused for you? What do you fear most about your sickness?
	The LEARN Model (36, 57) (*N*=2)	Listen with sympathy and understanding to the patient's perception of the problem Explain your perceptions of the problem Acknowledge and discuss the differences and similarities Recommend treatment Negotiate treatment
	The BELIEF Model (57) (*N*=1)	Beliefs about health (What caused your illness/problem?) Explanation (Why did it happen at this time?) Learn (Help me to understand your belief/opinion.) Impact (How is this illness/problem impacting your life?) Empathy (This must be very difficult for you) Feelings (How are you feeling about it?)
	The Four Habits Model of Highly Effective Clinicians (36) (*N*=1)	Invest in the beginning Elicit the patient's perspective Demonstrate empathy Invest in the end

### Healthcare provider skills (N=32)

This theme encompasses the skills required for culturally competent communication. It addresses actions required for integrating cultural knowledge (5, 36) and knowledge of diverse population health into clinical practice (24). Effective communication skills (11, 18, 37) were most prominently featured in the included literature (*N*=18). Using simple language (18–42) and checking patient understanding of information given (36, 39–41, 43, 44) were the most cited communication skills.

Managing difference in the patient–provider encounter (*N*=13) can be challenging. The literature underscored that healthcare providers should avoid stereotyping and generalisations (5, 11, 37, 18, 36, 44, 45).

Skills related to building the patient–provider relationship (*N*=12) ranged from the significance of the initial medical encounter (11, 36, 46, 47) to specific relational skills like building rapport (41, 48), gaining patient trust (11, 17, 49), addressing patients appropriately according to their cultural preference (11), and engaging in culturally sensitive communication (50).

The importance of assessment skills were also underscored in the literature (18, 36) and specific assessment skills for conducting a patient assessment beyond the biomedical aspect (*N*=13) were highlighted (41, 46, 51).

Key findings pertaining to accommodating the patient's family (*N*=5) included communicating with the patient's extended family (11), investing in and gaining family trust (11, 38), balancing autonomy and dependency when meeting patient and family needs (52), and affording the family the maximum control possible (51). Accommodating religion and spirituality (*N*=4) by recognising patients’ spiritual needs (51), acknowledging religion in the patient's belief system (11, 42), and demonstrating respect for religious beliefs (38) were also identified as key findings.

### Healthcare provider awareness (N=24)

Cultural awareness is an essential part of delivering culturally competent patient–provider communication (46, 53). Contextual awareness (*N*=11) relates to variables such as the country's socio-political history (41), socio-cultural factors (45), patients’ phases of acculturation to the dominant culture (47), and patient demographics in the service area (38, 42, 48, 52, 54).

Self-awareness (*N*=9) (11, 12, 18, 36) with regard to the provider's own culture (55); cultural beliefs (19); health belief systems (54); spirituality (51); and cultural assumptions, personal biases, and stereotypes (5, 19, 45, 54, 55) is supported by various authors.

Interpersonal awareness (*N*=5) with regard to inherent power differentials between healthcare providers and patients (41), the interaction between patients and healthcare providers’ cultures during the medical encounter (40, 55), and communication differences between cultures (36, 48) was highlighted in the literature.

Awareness of cultural expectations in the healthcare setting (*N*=5) pertains to the level of family involvement required (1, 44, 52, 54, 56) and the degree of direction expected from healthcare providers which may be more than what typically predominates in Western settings (54).

### Healthcare provider knowledge (N=22)

This theme (*N*=22) highlights the acquisition of sound factual knowledge and an understanding of various cultural aspects (11). When obtaining this culture-specific knowledge, healthcare providers should be cognizant of intra-cultural differences (5, 36, 44, 52, 57).

Context-specific knowledge (*N*=9) relates to knowledge of cultural groups seeking services in the provider's clinical setting (11, 18, 36, 47, 52, 58).

The importance of healthcare providers’ self-knowledge (*N*=6) pertaining to their own culture (11, 18, 36, 59), belief system (18), and biases and stereotypes (5, 11, 18, 54) is emphasised.

Similarly, knowledge of patients’ cultures (*N*=5), including their health belief systems (11, 39, 44), their traditional health systems (44), their processes of decision-making (1, 44, 47), and their standards of etiquette (1, 44), is underscored in the literature.

Knowledge of the broader contextual variables (*N*=5) centres on the socio-political barriers to accessing healthcare (11), the socio-historical cultural context and its influence on patients’ and families’ view of cancer (5), and the socio-cultural differences between the self and patient and its impact on patient–provider communication (18).

### Culturally competent healthcare systems (N=22)

Culturally competent communication extends beyond the individual provider to the healthcare system. Culturally competent healthcare systems are agents for the provision of appropriate patient care for diverse population groups that extend beyond addressing individual patient needs, to policy and community level (5, 37, 39, 43). Specific organisational strategies for culturally competent communication are well-represented in the literature. The most common strategies were the use of patient navigators (11, 24, 47, 48, 60, 61) and professional translators (1, 5, 11, 39, 41, 44, 45, 48, 54, 56, 57, 59).

### Healthcare providers’ personal characteristics and attitudes (N=13)

This theme highlights healthcare providers taking responsibility for cultural aspects of health and illness, and for combating discrimination in healthcare settings (45). The literature provided an extensive list of healthcare provider personal characteristics and attitudes that can facilitate culturally competent communication which is featured in Table 2. The most prominently featured healthcare provider attitude pertained to demonstrating respect for cultural diversity and patients’ cultural values (5, 11, 39, 42, 45, 52, 54, 62).

### Models of effective cross-cultural communication (N=3)

Models of effective cross-cultural communication (*N*=3) have been cited in some of the documents included in this integrative review. Kleinman's questions (17, 57), the LEARN Model (36, 57), the BELIEF Model (57), and the Four Habits Model of Highly Effective Clinicians emerged as key findings with regard to this theme.

## Discussion

The aim of the integrative review was to determine how culturally competent patient–provider communication is best delivered to adult patients diagnosed with cancer. Several important themes emerged about how this can be achieved. Despite the exhaustive nature of the integrative review a number of limitations remain. Only databases available at the university where the searches were conducted were used. Interlibrary loans were then used to obtain other documents. Two key documents could not be used because they could not be obtained by the university libraries. Most of the documents have been evaluated as level VII evidence (*N*=25), the lowest level of evidence. Eight of the documents fulfil the criteria for level VI evidence, and only two of the documents could be evaluated as level IV evidence.

There are a number of possible reasons for the lack of research at higher levels of evidence. The concept of cultural competence first appeared in the social work and counselling psychology literature in 1982 (30). A report issued by the US Department of Health and Human Services in 2001 highlighted that despite widespread policy recognition of the important role that cultural competence plays in facilitating accessible and effective healthcare for culturally diverse populations, policymakers were still in the early stages of defining cultural competence in a manner that facilitates empiricism and implementation (63). This lack of consensus on defining this concept was apparent in this report almost two decades after the concept first appeared in the literature. More recent literature still reports that despite the proliferation of cultural competency frameworks and models since their inception, there is still no one authoritative framework available (64, 65). There are a number of consonant concepts available such as culturally appropriate care, culturally sensitive care, and so forth, which further complicate the cultural competence theoretical and applied landscape (30, 64). A lack of uniformity in policy making with regard to comprehensive versus specific approaches to cultural competence has resulted in a burgeoning of ideas and methodologies about what constitutes cultural competence (63). The literature also indicates a lack of agreement on how best to implement cultural competence (65), and research on interventions for improving cultural competence in healthcare tends to lack methodological rigour (64). Hence, despite the recognition of how beneficial cultural competence can be in rendering effective healthcare services to diverse population groups, the lack of uniformity on conceptual, intervention, and policy fronts results in a myriad of disparate information. It is therefore hypothesised that while there is extensive literature on cancer health disparities and use of ‘cultural competence’ as a means of addressing these disparities (25, 66–68), research on how best to deliver culturally competent patient–provider communication to patients diagnosed with cancer is sparse owing to the aforementioned challenges associated with the concept of cultural competence.

Despite these challenges, the results of this integrative literature review provided useful insights for clinical practice. Engaging in culturally competent communication requires ‘communicating with awareness and knowledge of healthcare disparities and understanding that socio-cultural factors have important effects on health beliefs and behaviours, as well as having skills to manage these factors appropriately’ (20). The first three themes clearly illustrate this definition. The personal characteristics and attitudes required for culturally competent communication also emerged from the literature. Furthermore, the findings extend from the individual provider to emphasising culturally competent healthcare systems and models for culturally competent communication that can guide practice. The literature highlights the importance of this extension by emphasising that cultural competence should be addressed at policy, organisational, and systems levels (41). The information was categorised into various themes and subthemes to facilitate ease of reference and application in clinical practice. However, the findings related to these themes should be treated with caution as the results are based mostly on low-level evidence (Level VII) (32), indicating the lack of research using methodologies linked to high levels of evidence in this study area. In addition, all the studies were international and only one of the studies focused on an African refugee population albeit in the context of the US. The unique African setting necessitates and could greatly benefit from research on culturally competent patient–provider communication at higher levels of evidence.

## Conclusions

The findings of the integrative literature review have important practice implications. The themes that emerged during the integrative review process provide some insight into the ‘how’ of delivering culturally competent patient–provider communication to adult patients diagnosed with cancer. The grave need for scientifically rigorous research yielding higher levels of evidence in the field of cancer and culturally competent patient–provider communication is emphasised by the lack of quality evidence for all the themes that were presented in this integrative literature review.

## Supplementary Material

Culturally competent patient–provider communication in the management of cancer: An integrative literature review

## Paper context

Patient–provider communication in cancer care as well as cross-cultural clinical settings is known to be challenging. This article provides information on how healthcare providers can deliver culturally competent care to cancer patients when working in cross-cultural clinical settings. The integrative literature review was performed to explore existing evidence and revealed that more rigorous research yielding higher levels of evidence is needed in the field of culturally competent patient–provider communication with cancer patients.
