# Supplementary material for: Culturally competent patient–provider communication in the management of cancer: An integrative literature review
Source: Glob Health Action. 2016 Nov 30;9:10.3402/gha.v9.33208. doi: 10.3402/gha.v9.33208 (PMC5134830; doi:10.3402/gha.v9.33208)
Supplement: Culturally competent patient–provider communication in the management of cancer: An integrative literature review [file GHA-9-33208-s001.doc]

Supplementary File: Summary of 35 Included Records

| **Author and Year of Publication** | **Type of Study** | **Summary of Findings** | **Level of Evidence** |
| --- | --- | --- | --- |
| Atkin et al. 2014 | Qualitative study | Practitioners, despite a commitment to sensitive care, struggle to reconcile individual behaviour with what they think they know about South Asian cultures which creates misunderstandings, leading to poor practice. Strategies for cultural competence: self-awareness; awareness of cultural practices; demonstrating sensitivity; integrating cultural competence into clinical practice; challenging beliefs about cultures. | Level VI |
| Barclay et al. 2007 | Literature study | Reported on the following strategies: awareness of a possible need for the provider to take a more directive role, of patient demographics in service area, of own health belief system and own cultural biases; accommodating patients that require family involvement; knowledge of own cultural biases. Urged active exploration of cultural issues with patients and preferences for truth disclosure. Proposed demonstrating respect; recognising patients' health beliefs; and using professional translators. | Level VII |
| Beyene 1992 | Case study | Key strategies reported: understand and accommodate the role of the family; allow family participation in care if required by culture; do not misjudge level of acculturation with dominant culture; demonstrate warmth; be culturally sensitive to beliefs and practices; use professional translators. | Level VI |
| Chambers 2008 | Book chapter | Strategies reported included: awareness of dominant cultural narratives and of patients’ tendency to mix allopathic and traditional medicine; knowledge of own culture; knowledge of the community’s clinical experience; openness about own cultural frame; identify redressive actions in a community; use professional translators; locate and use cultural informants. | Level VII |
| Chaturvedi et al. 2014 | Case study | Highlighted importance of awareness of patient education levels and the role of culture in health belief systems. Emphasised that culture shapes patients’ interaction with the healthcare system. Strategies included: effective communication; explore patient and family perspectives; respect religious beliefs; enhance patient trust; treat patients equally; simplify information. | Level VII |
| Cohen & Palos 2001 | Literature study | Defined key concepts and summarised available guidelines to assist with providing culturally competent nursing care. Concluded that becoming culturally competent begins with understanding terms and concepts that are essential in developing cultural awareness, knowledge and skills. Emphasised sensitivity to language and the history of the development of some labels, assessing communication needs and conducting cultural assessments. | Level VII |
| Coughlin 2014 | Literature study | Reported on the roles of cultural competency, patient trust, and health literacy in the oncology setting. Highlighted the need for culturally competent lung cancer patient navigators from the point of diagnosis to the initiation and completion of treatment, including cancer staging. | Level VII |
| Dein 2006 | Book chapter | Emphasised the importance of: awareness of own culture, interaction of patient and provider culture, own stereotypes; providing culturally and linguistically sensitive services. | Level VII |
| Die Trill & Holland 1993 | Case study | Cancer was used as a model to highlight major cultural issues that should be considered in order to increase cultural sensitivity in the medical setting: family function, sex roles, language, disclosure of disease-related information, pain, attitudes towards illness and health practices, immigration, region, autonomy versus dependency, and death and bereavement. Emphasised avoiding cultural stereotyping when being culturally sensitive and being respectful. | Level VI |
| Epner & Baile 2012 | Case study | Argued that the categorical or multicultural approach to cultural competence results in stereotypical thinking. The cross cultural approach focuses on foundational communication skills, awareness of cross-cutting cultural and social issues, and health beliefs that are present in all cultures and offers a patient-centred alternative. Highlighted various models of effective cross-cultural communication and negotiation and the key elements of patient-centred care. | Level VII |
| Huang et al. 2009 | Qualitative study | Explored the social construction of cultural issues and found that previous experiences with people from other cultures and organisational approaches to culture and cultural care often influenced nurses’ views and understandings of culture and cultural mores and their beliefs, attitudes and behaviours in providing cultural care. | Level VI |
| Kagawa-Singer et al. 2010 | Literature study | Proposed strategies for reducing heath disparities in cancer care included: acknowledging own personal assumptions and biases; gaining an understanding of the cultural meaning of cancer; recognising culture of both patient and provider; awareness of cultural variations within cultures; integrating cultural knowledge into communication; providing information in a respectful manner to the designated receiver; using culturally appropriate nonverbal communication etiquette; engaging in congruent verbal and nonverbal communication; integrating community resources into cancer care; using professional translators; providing ethnic-specific services. | Level VII |
| Kagawa-Singer 2013 | Book chapter | Proposed cultural competence as a critical skill set for improving quality of health care, improving equity in the availability of healthcare and eliminating health disparities among population groups with regard to cancer care. Defined culture and cultural competency and discussed culturally based communication strategies to facilitate building trust between patient, family and provider with a view to negotiating mutually agreed upon goals for treatment. | Level VII |
| Kreps 2006 | Literature study | Provided an overview of cancer-related health disparities in the US and outlined communication strategies for reducing health disparities: minimise bias; demonstrate respect; make patients active participants; sensitive and adaptive communication to overcome health literacy barriers; use appropriate language; check patient understanding; use culturally sensitive print, audiovisual and electronic communication; formulate and implement policies to support effective cross-cultural communication. | Level VII |
| Lavizzo-Mourey & Mackenzie 1995 | Literature study | Acknowledged the critical role of culture and the importance of establishing guidelines for culturally competent medical care. Advocated having knowledge of: health-related cultural factors; the incidence and prevalence of diseases in the population; treatment outcomes peculiar to that population. Cautioned against blanket racial classifications; advocated integrating knowledge of diverse population health into clinical practice and converting an awareness of disease prevalence into practices and policies. | Level VII |
| Lichtveld et al. 2012 | Book chapter | Focused on the role of cultural competence in addressing health disparities and proposed strategies for creating a culturally competent cancer workforce. Highlighted being aware of phases of acculturation and advocated knowledge of: the patient’s culture, ethnic and geographic differences; processes of decision-making in different cultures; role of culture in health belief systems. Emphasised the significance of first medical encounters and the role and effectiveness of patient navigators. | Level VII |
| Longo & Slater 2013 | Case study | Illustrated the challenges of providing culturally-competent. Proposed strategies for culturally competent care included: recognition of spiritual and cultural needs; understanding that patients and families may respond differently to the dominant cultural expectation; conducting cultural and spiritual assessments of patients and families as part of the psychosocial assessment; maintaining open communication; providing family with maximum control possible; maintaining hope. | Level VII |
| Matthews-Juarez & Juarez 2011 | Literature study | Proposed practices for reducing health disparities and improving cultural competence included: awareness of personal biases and stereotypes; cultural knowledge of health-related needs; knowledge of own belief system, own culture and patients’ culture, own stereotypes and biases, cultural variations within cultures; knowledge of the impact of sociocultural differences on patient-provider interactions; effective assessment and communication skills; responsiveness to individual needs; effective patient-centred communication; respect for cultural differences; need for policies. | Level VII |
| Mitchell 1998 | Literature Study | Reported on the cross-cultural aspects of cancer disclosure. Strategies: awareness of culturally constructed myths about cancer; explore patients’ disclosure preferences before diagnostic testing; ensure that MDT is aware of disclosure preferences; be sensitive regarding terms used when discussing cancer. | Level VII |
| Moore et al. 2012 | Quantitative study | Found that: interpersonal treatment explained the greatest amount of patient satisfaction; adequate communication that addresses relevant patient needs and concerns during all medical encounters improves patient satisfaction; patient-centred care can build trusting relationships. Recommended respectful communication. | Level IV |
| Mullin et al. 1998 | Case study | Focused on cross-cultural patient-provider difficulties and how this interfered with quality of care. Recommendations to improve quality of treatment in cross-cultural settings included learning the patient’s language or developing a vocabulary of terms that are familiar to patients, not stereotyping, using professional translators. | Level VII |
| Muñoz-Antonia 2014 | Conference paper | Proposed that culturally competent care should become the mandate of all providers and specifically urged oncology providers to become more familiar with disease patterns and cultural health belief systems that impact cancer care. Highlighted awareness of cultural differences, communicating in an understandable manner and demonstrating respect. | Level VII |
| Murphy et al. 2010 | Literature study | Communication between provider and patient is proposed as important for reducing cancer care disparities along the disease trajectory especially as research cited in the article demonstrated that there is a disconnect between how providers think they are interacting and how patients are perceiving the interaction. Specific strategy - patient navigation. | Level VII |
| Ngo-Metzger 2006 | Literature study | Reviewed the literature focusing on the following five domains of culturally competent care: patient-provider communication, respect for patient preferences and shared decision-making, experiences leading to trust or mistrust, experiences of discrimination, linguistic competency. Advocated culturally competent care as a strategy for reducing or eliminating ethnic and racial health disparities. | Level VII |
| Pârvu et al. 2013 | Qualitative study | Reported that participants had particular explanations and meanings of illness which sometimes negatively affected coping. Suggestions for culturally competent practice: awareness of personal biases and socio-cultural factors; knowledge of cultural approaches to illness and treatment; ability to determine key decision-makers, avoid generalizations. Providers should take responsibility for: cultural aspects of health and illness, combating discrimination in healthcare settings and be culturally sensitive and respectful of cultural values | Level VI |
| Pesquera et al. 2008 | Literature study | Reported on cancer health disparities in the US and the role of culturally competent care in reducing these disparities. Detailed strategies for improving cultural competence with specific reference to building cultural awareness, knowledge and skills are outlined. Discussed cultural competence standards and improving cross-cultural communication. | Level VII |
| Pierce 2008 | Literature study | Proposed culturally competent practice strategies included: being self-aware; having knowledge of different cultures, own culture, patient's culture, patient’s health belief system, socio-political barriers to accessing healthcare; recognising inherent power differentials; engaging the patient; communicating; demonstrating respect; gaining patient and family trust; avoiding stereotyping /generalisations; clear and accurate communication; communicating with the patient’s extended family; congruent verbal and nonverbal communication; gaining access to and using patients' natural support systems to help them; using a patient navigator. | Level VII |
| Rollins & Hauck 2015 | Case study | Described the impact of culture on delivering bad news to patients and proposed a patient-centred approach to delivering bad news. Reported that patients vary in the preferences for receiving bad news both within and across cultures. Authors combined the SPIKES and Kleinman’s ethnographic models and these models’ inherent strategies to address these preferences. | Level VI |
| Shahid et al. 2013 | Qualitative study | Reported on communication challenges between health professionals and Aboriginal people with cancer. Found that Aboriginal people are marginalised and mistrust the health system. Proposed improving communication by heeding language, communication style, use of medical terminology and cross-cultural differences in time. Strategies included: avoid medical jargon, repeat explanations, use professional translators, use images to explain cancer. | Level VI |
| Song et al. 2014 | Quantitative study | Found that socio-cultural factors are associated with patient–provider communication. Reported that providers need to be aware of patient education levels, engage in behaviours that enhance trust, treat patients equally, respect religious beliefs, and reduce the difficulty level of the information. | Level IV |
| Surbone 2004 | Editorial | Focused on culture and cultural competence in oncology. Defined culture and argued the important role of culture in defining our identity. The complexity of cultural competence and the acquisition of knowledge, skills and attitudes to facilitate effective cross-cultural communication in clinical settings were argued. | Level VII |
| Surbone 2008 | Literature Study | Emphasised the role of culture in oncology communication. Proposed that cultural competence can improve therapeutic outcomes and decrease health disparities. Outlined the knowledge, skills, cultural aspects providers need to be aware of and attitudes required in cross-cultural oncology settings. | Level VII |
| Surbone 2010 | Editorial | Emphasised cultural preferences for truth-telling and the changing landscape in this regard. Proposed cultural competence as a skill set that reduces the likelihood of cross-cultural misunderstanding; that enables negotiation of mutually acceptable goals of treatment with patients and families cross-culturally, and facilitates relationships built on trust and mutual respect. | Level VII |
| Surbone & Baider 2013 | Case study | Discussed the ethical value and issues that can arise in the cross-cultural medical setting such as diverse attitudes and practices of truth-telling, family roles, end-of-life issues and caregiving practices. Defined and discussed cultural competence and its components. | Level VI |
| Thomas et al. 2010 | Book chapter | Proposed communication strategies included: be aware of nonverbal communication differences, gender, religious issues; build trust; build rapport; use print or visual media; attend to the patient’s expectations, feelings and concerns; make patients active participants; give clear information; encourage questions; use professional translators; use patient navigators. | Level VII |
